# Supplementary figures and images for: Ant visual route navigation: How the fine details of behaviour promote successful route performance and convergence
Source: PLoS Comput Biol. 2025 Sep 10;21(9):e1012798. doi: 10.1371/journal.pcbi.1012798 (PMC12445746; doi:10.1371/journal.pcbi.1012798)

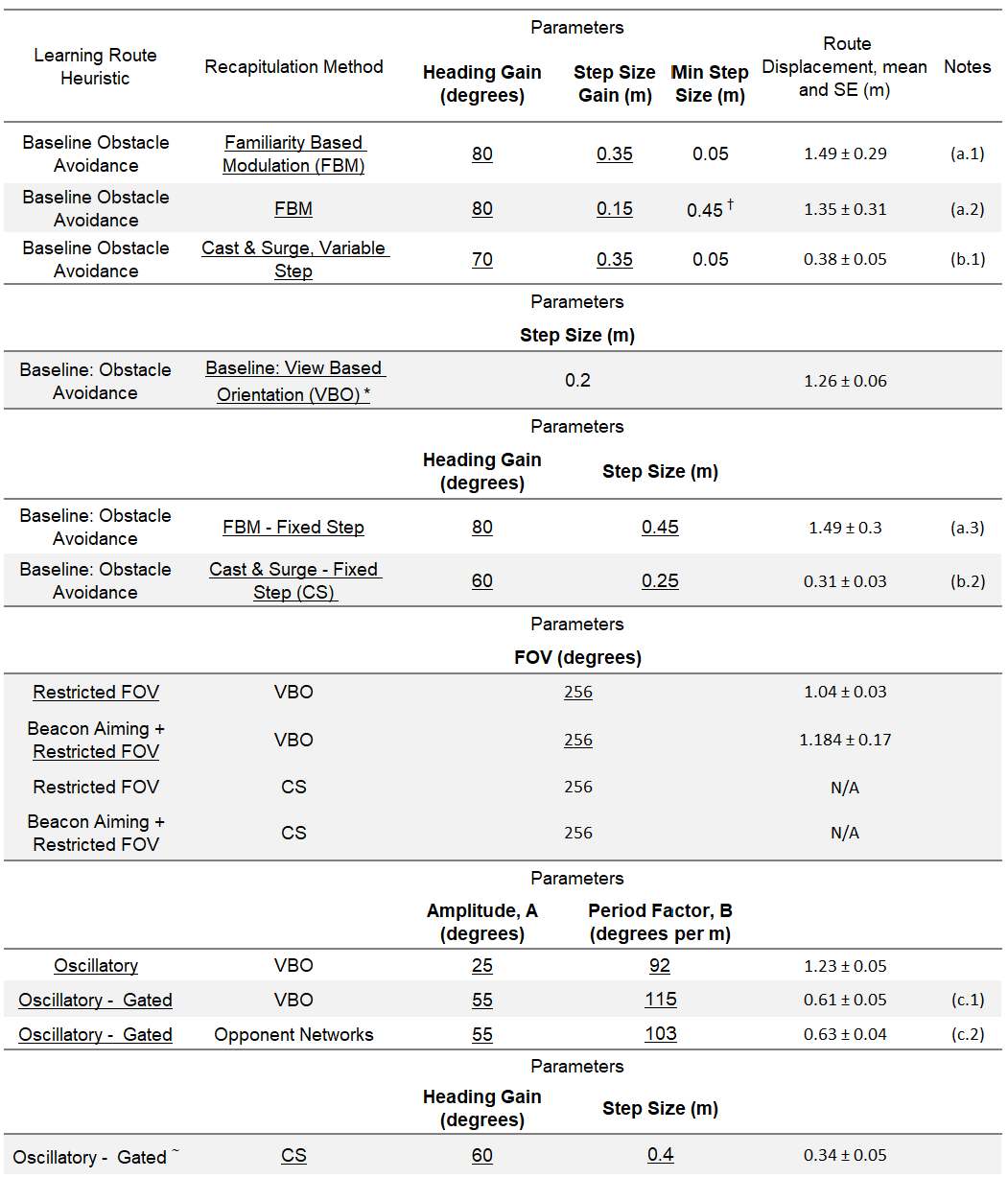

Supplement: S1 Table — Highlighted rows indicate strategies used in the main work. Underline denotes the method to which parameters in the same row correspond. Notes: (a) In familiarity based modulation (FBM) no significant difference between having a fixed step or a modulated step (one-way ANOVA, p = 0.93). For method consistency with [76] and for computational savings due to a higher minimum step, (a.2) is retained for the rest of this work. (b) For cast and surge, a modulated step size is considered, computed as sC=β(1−m(x,ϕ)), however, no statistically significant difference was found between having a fixed or modulated step (Wilcoxon signed rank test, W = 5, p = 0.625), for implementation simplicity (b.2) was retained. (c) When the training route is oscillatory and the view acquisitions are gated, there is no statistically significant difference (Wilcoxon signed rank test, W = 7, p = 1) in performance between using a singular network operating under VBO, or two networks trained on opposing views, for implementation simplicity and computational savings (i.e. one network as opposed to two) method (c.1) is therefore retained. Note that (c.1) has statistically significantly better performance compared to the oscillatory method which does not gate view acquisitions according to the period (0.61 ± 0.05 vs 1.23 ± 0.05, Mann–Whitney U = 0, p = 0.008). * Parameter derived from [109]. ∼ Oscillation Parameters set to those determined from parameter search for ‘Oscillatory Gated - VBO’. † minimum step size derived parameter search with fixed step. (PNG) [file pcbi.1012798.s001.png]

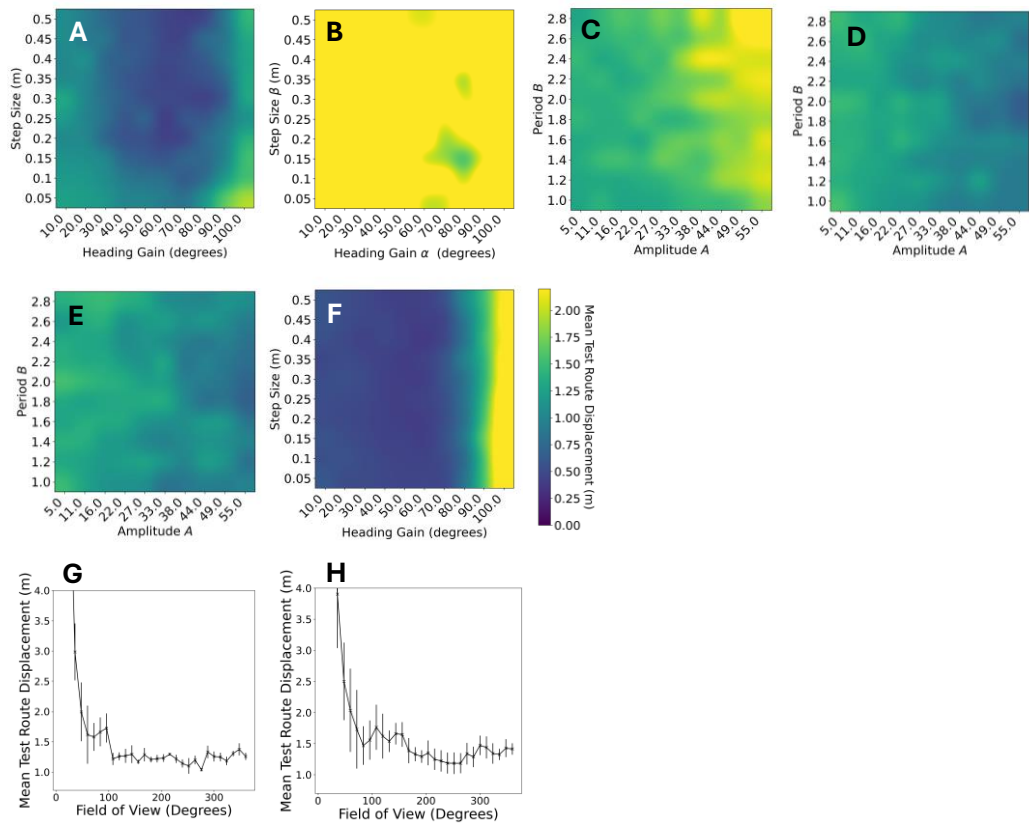

Supplement: S1 Fig — Heatmaps present grid searches across two parameters, totally 100 parameter combinations, selected going forward for those which minimised the mean test route displacement, evaluated for (A) Baseline (Obstacle Avoidance, OA) + CS (B) Baseline + FBM, minimum step = 0.45m (C) Oscillatory route (not gated) + VBO (D) Oscillatory route (gated) + VBO (E) Oscillatory route (gated) + Dual Opposing VBO networks and (F) Oscillatory route (gated) + CS. For the baseline VBO method, a parameter search determined that the field of view could be reduced with compromising convergence, but also does not enable it (G) Baseline + Restricted Field of View + VBO (H) Baseline + Beacon Aiming + Restricted Field of View + VBO, error bars represent standard error on the mean. (PDF) [file pcbi.1012798.s002.pdf]
