# Supplementary material for: Ant visual route navigation: How the fine details of behaviour promote successful route performance and convergence
Source: PLoS Comput Biol. 2025 Sep 10;21(9):e1012798. doi: 10.1371/journal.pcbi.1012798 (PMC12445746; doi:10.1371/journal.pcbi.1012798)
Supplement: Attachment — Submitted filename: ResponseToReview.pdf [file pcbi.1012798.s004.pdf]

Dear Editors,

We thank you and the reviewers for these constructive comments. Our detailed responses to each point raised are provided in red below.

Yours sincerely,

Amany Azevedo Amin

(Also on behalf of Professor Andrew Philippides and Professor Paul Graham)

# Response to reviewers' comments

## Reviewer #1

Minor revision:
1. Verification is almost sufficient to me. Just my curiosity, would it work well, in relatively new environments, e.g. like that in Figure 3 or 4, but test in a similar but different environment? Would be great to show or discuss on this aspect.

Route learning heuristics and recapitulation strategies are evaluated across 75 environment seeds, representing ‘similar but different environments’. This is stated in line 311 and the captions for Figure 1 and Figure 3, however we have now added a statement in the methods (line 834) for clarification.

2. Figure 1. Caption described the experiments, however, seems not enough to make it clear about the experiment setting and how the experiments have run, add one subplot to describe these schematically may help, or maybe consider swap the order to show those in Figure 2 first in Figure 1.

We now include a schematic figure describing the behavioural learning heuristics and the recapitulation strategies. We also add a methods figure to outline the experimental procedure.

With these changes made in mind, we would prefer not to swap the original figure 1 and 2 because we are reticent to comment on `typical’ behaviour presented in a single environment seed (i.e. submitted manuscript Fig. 2) *before* demonstrating the results gained through the systematic analysis across 75 seeds (as per submitted manuscript Fig. 1),

3. Minor issues:
Line 129, please explain VBO here.

While this section has now been reduced to address comments from reviewer 2, we ensure that VBO is explained in the introduction (Line 43)

Line 149, explain PI when first use, though noticed in line 157.
As per the suggestion from reviewer 4, what was previously referred to as path integration (PI) is more accurately described as a ‘global vector’ with respect to our simulations. We therefore change applicable instances to global vector throughout, which we do not abbreviate.

Line 854, weights are updated via, the equation (3) is only the change of weight, how to update weights may also need to explain.
Line 902: A formula for the application of the change of weight is now included.

# Reviewer #2:

**(1) The Introduction and the first sections of the Results are rambling, need to be shortened and made crisper. My main suggestion is to have a separate section on Models before the Result section and to add a figure in which the different learning and recall regimes that are being proposed and tested in simulation are clearly explained in the form of schematic diagrams.**

We have shortened the introduction and created a section on models which contains a crisper version of what was previously in the first section of results. We also include a new schematic figure to complement this (page 8) .

**(2) The Results section contains frequent elements of literature review, discussion and interpretation.**

We have chosen to use the PLoS CB permitted style of having detailed methods at the end of the manuscript. This allows for a narrative and context rich results section, with some interpretation throughout while saving major points for the Discussion. We have chosen this format because we want the paper to be accessible even for readers who do not need to know the fine methodological detail.

**(3) Throughout it remains unclear, how the 'oscillatory' models here relate to those proposed by Murray et al. 2020 and LeMoel&Wystrach 2020.**

The strategy of LeMoel&Wystrach & Murray et al is an extension of the strategy of Kodzhabashev and Mangan (Familiarity Based Modulation), which we cover comprehensively. In Kodzhabashev and Mangan, the turning amplitude of the agent is determined by the familiarity associated with the current view. In LeMoel&Wystrach & Murray et al, this amplitude is instead driven by the difference between attractive and opponent familiarity values associated with the current view. In both cases, no on-the-spot rotation is performed.

Murray et al exhibited some route following with a limited example in their Fig 1 E (3 paths, no displacements) and a comparison between their method and that of Kohzhabashev and Mangan in their Fig. 5. However, this comparison was only assessed qualitatively with respect to differences in behaviour around the goal area. No comparison was made with respect to route following quality and indeed the release points do not appear to be standardized between the two strategies (Fig. 5 A). While we cannot comment without statistics, the route following performance for the limited examples given that do share visually similar release points demonstrate a similar level of successful route following between their method and that of Kohzhabashev and Mangan.

In the work of LeMoel&Wystrach agents are subject to a training route, however neither route

following nor route convergence is demonstrated or discussed.  This work essentially presented a homing strategy for agents displaced radially with respect to the goal location, rather than for those requiring route travel to reach a destination.

It is important to note that successful homing strategies do not necessarily translate to route following (there is extensive work on this, summarised in Baddeley et al, 2011, Adaptive Behaviour) . However, given the robust homing performance demonstrated by the work of LeMoel&Wystrach, we already acknowledge (line 705-707 of the submitted manuscript) that their approach might be the basis of a more sophisticated goal-centered learning walk for the purposes of homing specifically.

As we see no evidence that these variants are an improvement on Kodzhabashev and Mangan for route *following*, we decided to consider the original strategy of Kodzhabashev and Mangan rather than these variants. This strategy was (a) demonstrated explicitly for route following and (b) stressed that ‘emergent’ search scans for finding familiarity occured when the agent was placed off-route, which could be construed as suggesting that convergence should follow.

The Cast and Surge approach here, as described in (what is now) Models and Methods, is a development on View Based Orientation (or the Visual Compass), where the on-the-spot scan is first performed (unlike Murray et al or LeMoel&Wystrach). When in casting mode, the agent aligns itself to the heading resulting from the on-the-spot scan and then sets a heading according to the associated *best* familiarity value, rather than the *original* familiarity (a variation on  Kodzhabashev and Mangan). It therefore most accurately bears resemblance with elements of the visual compass strategy and elements of the Kodzhabashev and Mangan strategy. We recognise that this was not clearly acknowledged when outlining the models and therefore we outline that this is, in part, a variant of their strategy in section ‘models’, subsection ‘cast and surge’ (lines 251 to 254) and in the discussion (line 680).

**Detailed comments**

**Line6: Maybe refer also to Huber & Knaden (2015) J Comp Physiol A (2015) 201:609–616 demonstrating very long PI guided trips.**
Now included.

**Line8: ‘in preference to PI’: Narendra et al (2013) Proc Roy Soc London B280: 20130683.**
Now included.

**Line19: reviewed in Zeil (2023) J Comp Physiol A 209: 499–514.**
Now included.

**Line36: ‘the current view’ does not ‘find a minimum of the rIDF’**.
Line 35: Rephrased to ‘(i.e when the agent has located the minimum of the rIDF)’

**Line 39: from behavioral analysis?**
Corrected

**Line55: Also refer to Hoinville&Wehner (2018) PNAS 115: 2824–2829.**
This paper is mainly about integration of guidance strategies and uses a very abstract approximation to view based orientation, rather than demonstrating an end to end implementation from vision to motor commands. Therefore we do not believe that it is relevant here.

**Line59ff: 'the best match…is an orientation parallel to the route…'. This is certainly true if the rIDF is dominated by distant visual features, but I am not sure whether this is also true in more cluttered environments?**
As per the references which immediately precede this statement, parallel route following (and therefore parallel headings) have been demonstrated across a range of simulated and real environments, including in the original work of zeil in 'outdoor scenes' and on board robots in cluttered indoor settings. We are principally concerned with ant-like habitats, and this work demonstrates parallel route following across 75 procedurally generated environments with a feature density selected to match a known ant habitat. Investigating how view based orientation performs across different environment densities is beyond the scope of this work but might be explored in a future project. However, we will note that clutter is a question of scale: we expect a smaller agent in a cluttered environment to exhibit the same behaviour (within a smaller range) as a large one in a sparse environment.

**Line69: Also quote Narendra (2007) J Exp Biol 210:1804–1812 here?**
Line 72: Now included

**Line81: 'enables future needs for convergence'?**
Line 83: Changed to 'promote route convergence'

**Line82: What is a 'situated' approach?**
Line 84: Expansion of the term 'situated' has now been inserted

**Line86ff: This whole section from 'Specifically we …' to '…during learning and recapitulation' should be significantly shortened. It is unnecessary and in places badly worded (see examples below). Without the details of your different model assumptions, it is very confusing.**
**Line88: 'exemplars of view based orientation type recapitulation strategies'. Find a nicer way of saying this…**
**Line90: 'leads to parallel route following after sideways displacements'?**
**Line97: 'what is a learning loop heuristic'? Just try plain language.**
**Line102: What is VBO?**
In response to all comments relating to lines 86 to 109, we agree that many elements of this section were superfluous and confusing. This section has now been significantly shortened.

**Line110-line313: This section does not present Results, but a description of Methods and Models. I appreciate that much of it is needed to assess your results, but I suggest to move it to a different heading after the Introduction and before Results, to de-wordify it and to help readers with a figure that explains your different learning and recapitulation schemes with schematic diagrams.**
We have relocated this to a 'models' section and de-wordified with the assistance of a schematic figure (page 8).

**Line119: '…paths converge back to the training route after displacements perpendicular to the route'?**
Lines 110-112: We have rephrased this sentence to acknowledge the perpendicular displacements.

**Line129: Here's the VBO again. Unexplained…**
Corrected

**Line143-145: Cut.**
Done

**Line147-line156: Cut.**
Done

**Line166: What is 'inherent structure'?**
This attempted to explain that while routes will take a different form, they follow the same 'rules'. However, we see that this is superfluous and have now streamlined this sentence.

**Line192ff: 'they are subject to …'. Unless they are tagged with the 'home' direction, like in the suggestion made by Jayatilaka et al. (2018) J Exp Biol 221: jeb1885306; Murray et al (2020) J Exp Biol 223: jeb210021 and Le Moel & Wystrach (2020) PLoS Comput Biol 16(2): e1007631, that ants may memorize both attractive and repellent views?**
Lines 148 to 165: We now add a couple of paragraphs discussing these works and outline why we did not pursue this strategy.

**Line194: What is a 'single' ….model?**
Line 163: We have now clarified by stating 'single network'. The references stated above require multiple models, to encode for instance, attractive and repellent views.

**Line210: Your references do not cover wasps. If you want to include them, refer to Stuerzl et al (2016) Curr Biol 26: 470-482.**
Rectified

**Line211: 'repeatedly departs from and returns to the site of interest'. That is true for learning walks and the exploration flights of honeybees and bumblebees, but not for the initial part of learning flights.**

Line 182: We have clarified this sentence as an example of the behaviour in ants.

**Line226: IDFs haven't been defined yet.**
IDFs, rIDFs and tIDF were defined in lines 20 to 23

**Line240: 'in silico' rotation.**
All instances corrected

**Line265ff: I do not understand these statements regarding scanning. First (1), I am not aware of many systematic video-based analyses of scanning along routes and second (2), why does a 'method which relies only on the current view' not require frequent scanning'?**

*(1)* Added reference to *Wystrach et al. (2014) J Comp Physio 200: 615-626* and described the observed frequency of on-the-spot scans (i.e. during uncertainty)

*(2)* Lines 231-238: Clarified that this section relates to on-the-spot scans. Also clarified the distinction between using the established VBO method (frequent on the spot scans for generation of an rIDF) and using the familiarity of the current view alone to determine the next step.

**Line274ff: First, how does this 'Cast and Surge' strategy relate to LeMoel&Wystrach's and Murray et al's algorithm of attractive and repellent views driving the scanning amplitude? And second, does the 'Cast and Surge' algorithm explain the observation that ants close to the goal increase scanning amplitude and changes in direction (e.g. Murray et al. (2020) and Zeil (2025) J Exp Biol 2288: jeb249499)?**
1) Please see the main statement above

2) lines 695-697: Yes, it is a fair point that the reduction in familiarity associated with views approaching the goal and beyond could indeed be an explanation of this behaviour observed in ants. We now include a reference to Murray et al and Zeil when commenting on this behaviour in the discussion.

**Line314: This is the first time now that you present 'Results'. It would be easier for the reader to understand all this if you presented Fig. 2 first, followed by the quantification in Fig. 1.**
Now that we include a schematic (page 8) to explain the models in a separate 'Models' section, and a separate Methods figure with a schematic illustrating the experimental procedure, we wish to retain the ordering of these figures in 'Results'. This is because we are reticent to comment on `typical' behaviour presented in a single environment seed (i.e. previous Fig. 2, now Fig 3) *before* demonstrating the results gained through the systematic analysis across 75 seeds (as per previous Fig. 1, now fig 2)

**Line342ff: There are quite a few examples such as this, where you mix methods, results, interpretation & discussion…**
As per the response above, the models have been removed into a separate section. This

particular sentence does include a minor point of interpretation/ discussion. However as stylistic choice we save the major and overarching observations of importance for the discussion. Please also see our response to comment (2) above.

**Line348: '…perform better elsewhere'. What do you mean? On Mars?**
Lines 320-322: Clarified relationship to spatial frequency

**Line381: '…which form the baseline…'**
Corrected

**Line421ff: Again a bit of discussion in the Result section.**
Please see response to line 342.

**Line455ff: What do you mean by 'potential complexity'? You seem to suggest that it is necessary to also record gaze directions, but a more serious 'complexity' may be to distinguish between guidance (how does familiarity drive behaviour) and sampling (the need for scanning)?**
Lines 429:430: We adjust this to better explain what we were trying to convey

**Line462ff: with 2J ect are you referring to Fig. 2J?**
Corrected

**Line496: '…used to train…'**
Corrected

**Line544: '…results are summarized…'**
Adjusted and streamlined.

**Line582ff:(1) I don't quite understand why full 360deg scans would be needed at all, except to get an initial bearing when displaced. (2) In cases where gaze directions of ants on familiar routes have been recorded, there is always an oscillation (e.g. Lent et al (2010) PNAS 107: 16348-16353; Zeil (2025)).**

(1) We agree, but this is the approach taken by many works (e.g. Baddeley et al, 2012, PLOS Comp Bio; Knight et al, 2019, Artificial Life). In addition to this, any works using a reduced range did not necessarily justify this (e.g. 296 deg in Ardin et al, 2016, PLOS Comp Bio; 200 deg in Gattaux et al, 2023, preprint). Therefore we start with the default on-the-spot scan range of 360 degrees, but show through our experiments that a maximum scan range of 160 degrees is sufficient (i.e. does not compromise route convergence).
(2) Lines 589- 594: We now include the later work of lent (2013, Current biology), as well as Zeil 2025 and Wystrach 2014, as examples of how this scan range modulation aligns with observed behaviour.

**Line589ff: Is this not similar to Murray et al (2020) and LeMoel&Wystrach (2020)?**

Please see the response to the statement relating to these works in the overall comments.

**Line605ff: What is the 'observed ant behaviour' you want to 'align' with?**
Line 589: We include a quote and reference to *Wystrach et al. (2014) J Comp Physio 200: 615-626,* and make this the focus of the statement.

**Line613ff: '…without and with scanning…'. Fig 4C is without and Fig. 4D is with modulation. Line** Line 601: Corrected

**Line689ff: Again, is this not very similar what Murray et al (2020) and LeMoel&Wystrach (2020) propose?**
Please see the response to the statement relating to these works in the overall comments.

**Line698ff: '…as casts increase around the goal location'. This is exactly what is observed in ants (Murray et al 2020, Zeil (2025) J Exp Biol 228: jeb249499).**
**Line707: Refer in addition to Murray et al 2020 here.**
Line 696: We now include these references.

**Line723: Are there good reasons why you don't refer to Hoinville&Wehner (2018) in this context?**
Line 713: We now include a reference to Hoinville&Wehner to acknowledge their work on integration of belief vectors into a useful 'sensory' vector.

**Line796: You may want to refer to more recent work on honeybee/bumblebee exploration flights by Degen et al and Woodgate et al (2016).**
Line 822: Reference now included

**Line797: There are data showing that learning walks can be much more extensive: Deeti&Cheng(2021) J Exp Biol 224: jeb242177.**
Line 823: Adjusted and reference included.

**Line811ff: tIDF catchment areas depend on the depth structure of the habitat.**
Line 837: Corrected

**Line812ff: In principle, can 'cast and surge' be considered to be a simple (move and compare) gradient descent method?**
Line 838: Cast and surge is indeed a simple gradient ascent/ descent method, now explicitly stated

**Line827: It would be really interesting to check the degree to which the absence or presence of distant visual features affects your simulations. The rIDF would be more robust and would provide directional guidance over a larger range, if distant visual features were present.**
As the environment is populated with trees alone to beyond the agent's camera clipping

distance (set at 50m) there are no large distal cues such as, for example, mountains. This would indeed improve the rIDF, but not the tIDF. We agree that this is an interesting consideration and suitable for future work.

**Line837ff: (1) Views don't just 'appear'. Further down it becomes clear that learning is paced to 5 views per metre (of path?), meaning that views are learnt independent of whether they have changed or not. (2) Another interesting future project: views are only learnt, when the scene has changed.**

(1) Line 892: Clarified that views are stored at regular intervals along the path
(2) We agree that this an interesting future project

**Line842: What does 'selected for similarity with previous work' mean?**
Line 866: Adjusted for consistency and explained that this view size is more than sufficient for the route lengths under consideration.

**Line935: Give references for this statement.**
Line 986: References inserted

Check references. Many are incomplete, species names are not italic ect ect.
We have now reviewed our references, ensuring complete information and italicized species names.

# Reviewer #3:

1. Centralized vs. Decentralized navigation systems: A debate in insect navigation research concerns whether these systems are centralized or decentralized. As I understand it, different insect species—such as fruit flies (Drosophila), desert ants, and bees—employ varying navigation strategies. Path integration, visual homing, and route following are influenced separately or jointly by multisensory information from subsystems including the global compass, local compass, odometer, and other cues. Given these disparities in navigation mechanisms across species, what are the authors' perspectives on how this study contributes to the broader understanding of insect navigation? How might the findings be contextualized within existing knowledge of these different species?

Lines 620-624: We expand the last section of the first paragraph of our discussion to give our perspectives on how our work contributes to the broader understanding of insect navigation.

2. Potential for Robotic Implementation: How significant is the gap between the observed navigational behavior in ants and its potential implementation in robotic navigation systems? Could the proposed mechanisms be directly applied?

Lines 685-688, 728-730: We have expanded the sections in the discussion to include statements relating to applying these mechanisms to robotics.

# Reviewer #4:

Detailed Comments and Questions :

- **Claim vs. Figures**

In the abstract, you state that the “most successful recapitulation method” is ‘cast and surge.’ However, based on your Figures (1A, 1C, 1D, 1F, 1G, and 1H), for a 20 m route, the **best** method appears to be “Gated Osc. + Goal Loop + VBO,” both for the entire path and the final 5 m. Moreover, you only tested “baseline VBO + baseline CS” for the 100 m route, so it’s hard to see whether “Gated Osc. + Goal Loop + VBO” would similarly outperform cast and surge at that longer distance, even if you explained it later why it doesn’t appear, this raised question.

In the abstract we clarify that cast and surge is the best *recapitulation* method, i.e. when there are no specific modifications to the learning route. We also clarify that the size of the `goal loop’ should be tuned to the degree of divergence. In the results themselves, section 3.3 (line 437), we clarify that the goal loop is specifically tuned to the level of divergence seen, and in section 3.4 (line 487-489), we clarify that we wish to investigate the theoretical range that a ‘goal loop’ would have to cover for longer routes (where there is more divergence).

- **Use of “Embodied” (Line 82, Page 11**

I am not fully convinced by the term “embodied” here, as your simulation agent doesn’t share the ants’ exact movement dynamics or “walking” mechanisms. You do capture high-level sensory and motor components, but LAL, and CX or other insect-specific motor circuits are not truly modeled. The sentence from Lines 82–86 (p. 11) might need rephrasing or clarifying, because “embodied” usually implies that the agent’s body and environment are tightly integrated in a biologically realistic way (in real world as well).

Line 84: This is a fair statement, for a better description of the approach we have removed ‘embodied’ and use only the term ‘situated’, along with an expanded explanation of this term.

- **Systematic Scan Claims**

You repeatedly claim (Lines 124, 263, 442, 580, 585, 606) that systematic scanning is not strictly necessary for navigation. However, it has been shown feasible in actual robot systems that include lateralized mushroom-body models (for example, in the preprints by Lu et al. [2024] and Gattaux et al. [2024]). You do highlight that modulating the scanning steps is a novel point in your present “one-memory framework,” which is indeed interesting. Still, please recognize this earlier robotics work showing that onboard scanning can be totally removed for route recapitulation using familiarity.

Line 564-568: We now recognise these works and justify our approach in the results.

Lines 748-773: We also now specifically discuss these works in an extended paragraph in the discussion.

Former line 124 was removed based on the suggestion of another reviewer.

Line 230, Former line 263, is a remark by the author of the original work rather than our own work, which we clarify.

- **Goal Reaching vs. Route Convergence**

Throughout the text (e.g., Lines 97, 351, 360, 390, 484), it seems you sometimes conflate route "convergence" with actual goal "reaching." How precisely does your agent determine it has arrived at the goal rather than simply crossing the goal's location and continuing past it? If you manually stop it, or rely on ground-truth knowledge (Line 167 references ignoring path integration at recapitulation), that should be spelled out. Otherwise, the paper should clarify it only addresses "route following" (and you terminate the simulation artificially), rather than fully "goal reaching."

Lines 277-285: We rely on ground truth knowledge for both the metrics of route convergence and goal reaching. Goal reaching is simply route convergence with respect to the last 5% of the route (ignoring all the path that precedes it), to recognise that some strategies might not be convergent to the route, but still reach the goal. We agree that outside of the methods it is not clear that these are separate metrics and clarify this at the beginning of the results.

- **Path Integration Simulation**

Could you clarify how path integration was simulated? Is it via a "central complex" model, or did you just use a global "omniscient pose" to compute the vector to the nest/goal? If it's the latter, it might be better to say you used a ground-truth or global vector, rather than a biological path-integration system.

It is the latter, therefore we have modified the text throughout to 'global vector'.

- **Environmental Density (Line 418)**

You chose 0.007 trees/m². Did you test denser or sparser environments to support the general claims about the model's performance, especially regarding route convergence?

Not in this work, however this tree density is derived from a lidar scan of the habitat of visually navigating *Myrmecia croslandi*. Changing density represents an interesting future direction exploring convergent route strategies to differing visual ecologies.

- **Tree Dimensions and Visuals (Fig 2)**

A 2 m trunk diameter and up to a 20 m canopy diameter is quite large—could you confirm it's realistic or just a simplification for the simulation? A quick screenshot from the agent's point of view along the route might help readers understand what the environment actually looks like.

Eucalyptus trees in Australia can indeed grow very large and beyond this scale. However, the simulation is in general a simplification, even if the trees are within an accurate scale. For example, only trees are present (no other vegetation or landscape features), the ground is homogenous, there is no attempt to model the dynamics of tree distribution etc. For transparency, we now state the height range of trees used in the simulation in the methods (lines 856-866). To help readers, we also include a methods figure with an agent view and an environment view.

- **Motor Noise (Line 321)**

Could you clarify what you define as "motor noise"? Is this the stepwise oscillation pattern, or a separate additive noise in heading? A discussion of how gait oscillation (and noise in the agent's phase) might affect the recapitulation performance would be valuable.

Lines 868, 984: We now clarify that this is a separate additive noise in the heading, in a rearranged section of the methods titled 'Simulation environment and agent characteristics'.

Lines 993-995: In the methods section titled 'Oscillatory learning route' we also clarify that the agent's knowledge of its oscillation is perfect, but that motor noise compensates for this to some degree by introducing views learned at spurious directions.

- **Goal Loop Introduction**

The first mention of "goal loop" in the text appears in Fig. 1, yet it is only fully explained by Line 462. It might help to define "goal loop" earlier so readers know what they are seeing in the figure.

Line 190: Now defined in the preceding section titled 'models'

- **t-Tests (Lines 603, 1044, 1046, 1051)**

A t-test assumes normality. Did you check if the data were approximately normal, or how many data points each test included? A short justification would help solidify the use of t-tests.

Lines 587, 1098, 1100, 1105: Thank you for this reminder and checking for this does indeed show that the use of the t-test is unjustified. We have now adapted our statistics to use Wilcoxon signed-rank and the Mann–Whitney U test where appropriate (i.e. paired and independent data respectively).

- **Discussion vs. Results (Line 497)**

The paragraph starting at Line 497 seems more suited to the Discussion than the Results, as it is largely interpretative rather than describing raw findings.

Agreed, and indeed this point is also made in the discussion. Removed from here and expanded slightly in the discussion.

- **Strategic Conflict (Line 506)**

You compare “Gated Osc. + Res. FOV + VBO” to “Res. FOV + FBO” and “Gated Osc. + VBO,” suggesting they conflict. Could you expand on why they might “conflict”? For instance, do the underlying assumptions or directional biases differ in a way that reduces synergy?

Lines 479-482: We expand on why we think these strategies might be in conflict.

- **Typos (Line 537, 571)**

There appear to be small typographical issues in these lines. A careful proofreading should fix these.

We rephrase line 537 and correct the typo in line 571.

- **“Best” Solution (Lines 555, 648)**

You repeatedly label “Gated Osci. + CS” as the best solution, but for a 20 m route, the data seem to favor “Gated Osc. + Goal Loop + VBO.” You later explain the 100 m route situation. This discrepancy could benefit from a clearer explanation of which method is “best,” for which route length and conditions.

Line 533, formerly 555: we clarify that this is the best of the strategies evaluated, but believe we have shown appropriate consideration to the ‘goal loop’ mechanism in the sentences which follow.

Line 639, formerly 648: we clarify that it is an improved *recapitulation* strategy

- **“Other Areas of Navigation” (Line 652)**

The term is a bit vague. Perhaps clarify if you refer to general navigation problems.

Line 643: We now clarify, expressing use of the same strategy across different types of sensory input.

- **Infomax Model Explanation**

Early in the paper, you might briefly remind readers that the Infomax network is used to learn the panoramic views and how that differs from simpler snapshot banks or spiking networks. A short overview in the Methods or Introduction could help non-expert readers.

Lines 46-50: We expand our explanation of the infomax network in the introduction to include a statement on the infomax network performing feature extraction and memorisation.

Lines 876-888: In methods, under route learning, we provide a brief overview of snapshot banks and spiking networks, and justify our use of infomax.

Line 99-100: We also remind the reader that infomax is used to learn the route views in the new 'models' section.

- **Line 726 Claim**

You state that oscillation is new in the route context, but other references you provided show that certain scanning or oscillatory movements can enhance visual navigation. Consider clarifying that your main novelty is applying it to route convergence specifically, not just to general navigation.

Line 724: True, we now clarify that it is a useful motif specifically for route convergence.

- **Infomax vs. Mushroom Body Model**

Could you say a bit more about the limitations of Infomax compared to a mushroom body model? Do you need a global familiarity representation ?

Lines 876-888: In justifying the choice of model in the methods, we now outline the main limitation of infomax and explain our reasoning for using it over a spiking mushroom body model.
