# Supplementary material for: Ant visual route navigation: How the fine details of behaviour promote successful route performance and convergence
Source: PLoS Comput Biol. 2025 Sep 10;21(9):e1012798. doi: 10.1371/journal.pcbi.1012798 (PMC12445746; doi:10.1371/journal.pcbi.1012798)
Supplement: Attachment — Submitted filename: second_review.odt [file pcbi.1012798.s003.odt]

This article highlights an interesting problem: the emergence of parallel routes under states of the art ant-inspired visual route-following algorithms. The authors use an Infomax neural network to learn and recapitulate routes in a simulated environment, with a tree density of 0.007 trees/m². They conduct several experiments to investigate how well a simulated agent converges onto its training route. The paper’s approach (treating learning and route-recapitulation methods distinctly) is an excellent way to dissect which elements drive convergence.
In my view, oscillatory movement, beacon aiming, visual-based orientation (VBO), and familiarity-based modulation (FBM), while known in visual route-following contexts, are nicely integrated here. Cast-and-surge is also not entirely new but is creatively applied to visual familiarity. The gated-oscillation (i.e., “gated oscillatory learning”) strikes me as the most original contribution, and combining these known methods to address the underexplored problem of route convergence is a valuable advance. The goal reaching statements, with goal loop approach which couples views toward the nest, is interesting but seems slightly overfitted for the route convergence question and may raise questions. Overall, the article is well written and the experiments are clearly presented. 
Detailed Comments and Questions : 
Claim vs. Figures
In the abstract, you state that the “most successful recapitulation method” is ‘cast and surge.’ However, based on your Figures (1A, 1C, 1D, 1F, 1G, and 1H), for a 20 m route, the best method appears to be “Gated Osc. + Goal Loop + VBO,” both for the entire path and the final 5 m. Moreover, you only tested “baseline VBO + baseline CS” for the 100 m route, so it’s hard to see whether “Gated Osc. + Goal Loop + VBO” would similarly outperform cast and surge at that longer distance, even if you explained it later why it doesn’t appear, this raised question. 
Use of “Embodied” (Line 82, Page 11
I am not fully convinced by the term “embodied” here, as your simulation agent doesn’t share the ants’ exact movement dynamics or “walking” mechanisms. You do capture high-level sensory and motor components, but LAL, and CX or other insect-specific motor circuits are not truly modeled. The sentence from Lines 82–86 (p. 11) might need rephrasing or clarifying, because “embodied” usually implies that the agent’s body and environment are tightly integrated in a biologically realistic way (in real world as well). 
Systematic Scan Claims
You repeatedly claim (Lines 124, 263, 442, 580, 585, 606) that systematic scanning is not strictly necessary for navigation. However, it has been shown feasible in actual robot systems that include lateralized mushroom-body models (for example, in the preprints by Lu et al. [2024] and Gattaux et al. [2024]). You do highlight that modulating the scanning steps is a novel point in your present “one-memory framework,” which is indeed interesting. Still, please recognize this earlier robotics work showing that onboard scanning can be totally removed for route recapitulation using familiarity.
Goal Reaching vs. Route Convergence
Throughout the text (e.g., Lines 97, 351, 360, 390, 484), it seems you sometimes conflate route “convergence” with actual goal “reaching.” How precisely does your agent determine it has arrived at the goal rather than simply crossing the goal’s location and continuing past it? If you manually stop it, or rely on ground-truth knowledge (Line 167 references ignoring path integration at recapitulation), that should be spelled out. Otherwise, the paper should clarify it only addresses “route following” (and you terminate the simulation artificially), rather than fully “goal reaching.” 
Path Integration Simulation
Could you clarify how path integration was simulated? Is it via a “central complex” model, or did you just use a global “omniscient pose” to compute the vector to the nest/goal? If it’s the latter, it might be better to say you used a ground-truth or global vector, rather than a biological path-integration system. 
Environmental Density (Line 418)
You chose 0.007 trees/m². Did you test denser or sparser environments to support the general claims about the model’s performance, especially regarding route convergence?
Tree Dimensions and Visuals (Fig 2)
A 2 m trunk diameter and up to a 20 m canopy diameter is quite large—could you confirm it’s realistic or just a simplification for the simulation? A quick screenshot from the agent’s point of view along the route might help readers understand what the environment actually looks like. 
Motor Noise (Line 321)
Could you clarify what you define as “motor noise”? Is this the stepwise oscillation pattern, or a separate additive noise in heading? A discussion of how gait oscillation (and noise in the agent’s phase) might affect the recapitulation performance would be valuable. 
Goal Loop Introduction
The first mention of “goal loop” in the text appears in Fig. 1, yet it is only fully explained by Line 462. It might help to define “goal loop” earlier so readers know what they are seeing in the figure. 
t-Tests (Lines 603, 1044, 1046, 1051)
A t-test assumes normality. Did you check if the data were approximately normal, or how many data points each test included? A short justification would help solidify the use of t-tests. 
Discussion vs. Results (Line 497) 
The paragraph starting at Line 497 seems more suited to the Discussion than the Results, as it is largely interpretative rather than describing raw findings. 
Strategic Conflict (Line 506) 
You compare “Gated Osc. + Res. FOV + VBO” to “Res. FOV + FBO” and “Gated Osc. + VBO,” suggesting they conflict. Could you expand on why they might “conflict”? For instance, do the underlying assumptions or directional biases differ in a way that reduces synergy? 
Typos (Line 537, 571) 
There appear to be small typographical issues in these lines. A careful proofreading should fix these.
“Best” Solution (Lines 555, 648) 
You repeatedly label “Gated Osci. + CS” as the best solution, but for a 20 m route, the data seem to favor “Gated Osc. + Goal Loop + VBO.” You later explain the 100 m route situation. This discrepancy could benefit from a clearer explanation of which method is “best,” for which route length and conditions. 
“Other Areas of Navigation” (Line 652) 
The term is a bit vague. Perhaps clarify if you refer to general navigation problems. 
Infomax Model Explanation 
Early in the paper, you might briefly remind readers that the Infomax network is used to learn the panoramic views and how that differs from simpler snapshot banks or spiking networks. A short overview in the Methods or Introduction could help non-expert readers. 
Line 726 Claim 
You state that oscillation is new in the route context, but other references you provided show that certain scanning or oscillatory movements can enhance visual navigation. Consider clarifying that your main novelty is applying it to route convergence specifically, not just to general navigation. 
Infomax vs. Mushroom Body Model 
Could you say a bit more about the limitations of Infomax compared to a mushroom body model? Do you need a global familiarity representation ?
